# A new species (*Begoniagiganticaulis*) of Begoniaceae from southern Xizang (Tibet) of China

**DOI:** 10.3897/phytokeys.187.75854

**Published:** 2021-12-30

**Authors:** Dai-Ke Tian, Wen-Guang Wang, Li-Na Dong, Yan Xiao, Min-Min Zheng, Bin-Jie Ge

**Affiliations:** 1 Shanghai Chenshan Plant Science Research Center, Chinese Academy of Sciences, 3888 Chenhua Road, Songjiang, Shanghai 201602, China Shanghai Chenshan Plant Science Research Center, Chinese Academy of Sciences Shanghai China; 2 Shanghai Key Laboratory for Plant Functional Genomics and Resources, Shanghai Chenshan Botanical Garden, 3888 Chenhua Road, Songjiang, Shanghai 201602, China Shanghai Chenshan Botanical Garden Shanghai China; 3 Xishuangbanna Tropical Botanical Garden, Chinese Academy of Sciences, Mengla, 666303, Yunnan, China Xishuangbanna Tropical Botanical Garden Mengla China; 4 Guangxi Key Laboratory of Plant Conservation and Restoration Ecology in Karst Terrain, Guangxi Institute of Botany, Guangxi Zhuang Autonomous Region and Chinese Academy of Sciences, Guilin 541006, Guangxi, China Guangxi Institute of Botany, Guangxi Zhuang Autonomous Region and Chinese Academy of Sciences Guilin China; 5 University of Chinese Academy of Sciences, Beijing 10049, China University of Chinese Academy of Sciences Beijing China

**Keywords:** Conservation status, molecular evidence, morphology, southern Tibet, taxonomy

## Abstract

*Begoniagiganticaulis*, a huge new species in Begoniasect.Platycentrum of Begoniaceae from southern Xizang (Tibet) of China, is described. Morphologically, it is mostly similar to *B.longifolia* and *B.acetosella*, but clearly differs from the former mainly by its dioecious and taller plants, sparse hairs on abaxial veins, longer inflorescence, unique shape of fruits, and differs from the latter mainly by its late and longer flowering time, 6-tepals of female flower and 3-loculed ovary. The phylogenetic analyses also support the separation of the new species from other taxa. Based on the current data, its conservation status is assigned to Endangered (B2a) according to the IUCN Red List Categories and Criteria.

## Introduction

Zangnan (southern Tibet) of China is located to the south of the Himalayas, including most parts of Cona, Lhünzê, Mêdog and Zayü counties, and some smaller parts of Nang and Mainling counties (Liu 2019). This region is very warm and rainy because of the southwest monsoon carrying heavy water and heat from the Indian Ocean. Owing to high average annual precipitation and high-proportion of forest coverage (Hao et al. 2010), the plant diversity is very high in Zangnan. However, this area still remains under-explored and needs more study in the future.

After a series of plant surveys recently, the authors have a better understanding of the diversity of *Begonia* in Tibet, particularly in its southern part (namely Zangnan) including Mêdog county. Up until now, 39 species and 4 varieties had been found in Tibet (Gu et al. 2007; Camfield and Hughes 2018; Tian et al. 2020) (Table 1). In addition, *Begonialimprichtii* Irmsch. (Irmscher 1922) was newly reported by Borah et al. (2021a) in southern Tibet, but this record is likely based on a wrong identification and further study is needed. Of these, 31 species and 3 varieties are distributed in Mêdog. Recently, after several field surveys in Mêdog, we found several new species and at least three natural hybrids. Here we described *Begoniagiganticaulis* D.K.Tian & W.G.Wang sp. nov. from Mêdog, a new species of huge plant size, which is morphologically similar to both *B.longifolia* Blume (Blume 1827) and *B.acetosella* Craib (Craib 1912). The morphological differences of the three species are compared, and the new species is also supported by molecular evidence.

**Table 1.** A checklist of *Begonia* species in Tibet.

Species	Reference	County
*Begoniaaborensis* Dunn	Dunn 1920	Mêdog
*Begoniaacetosella* Craib	Craib 1912	Mêdog
*Begoniaannulata* K.Koch	Koch 1857	Mêdog
*Begoniaasperifolia* Irmsch.	Irmscher 1927	Bomê, Zayü, Lhünzê, Mêdog
*Begoniaburkillii* Dunn	Dunn 1920	Mêdog
*Begoniacathcartii* Hook.f. & Thomson	Hooker 1855	Zayü
*Begoniadioica* Buch.-Ham. ex D.Don	Don 1825	Dinggyê
*Begoniadifformis* (Irmsch.) W.C.Leong, C.I Peng & K.F.Chung	Leong et al. 2015	Mêdog
*Begoniaflagellaris* Hara	Hara 1973	Gyirong, Nyalam
Begoniaflavifloravar.flaviflora Hara	Hara 1970	Mêdog
Begoniaflavifloravar.gamblei (Irmscher) Golding & Karegeannes	Golding and Karegeannes 1984	Mêdog
*Begoniagiganticaulis* D.K.Tian & W.G. Wang sp. nov.	In this study	Mêdog
*Begoniagrandis* Dryand.	Dryander 1791	Zayü
*Begoniagriffithiana* (A.DC.) Warb.	Warburg 1894	Mêdog
*Begoniahandelii* Irmsch.	Irmscher 1921	Mêdog
*Begoniahatacoa* Buch.-Ham. ex D.Don	Don 1825	Mêdog, Cona
*Begoniairidescens* Dunn	Dunn 1920	Mêdog, Zayü
*Begoniajosephii* A.DC.	de Candolle 1859	Cona, Dinggyê, Lhünzê, Mêdog, Yadong
*Begoniakekarmonyingensis* Taram, D.Borah & M.Hughes	Taram et al. 2021	Mêdog
*Begonialabordei* H.Lév.	Léveillé 1904	Zayü
*Begonialimprichtii* Irmsch.*(wrong identification, based on the distribution and morphological characteristics)	Borah et al. 2021a	Mêdog
*Begonialongifolia* Blume	Blume 1827	Mêdog
*Begoniamedogensis* J.W.Li, Y.H.Tan & X.H.Jin	Li et al. 2018	Mêdog
*Begoniamegaptera* A.DC.	de Candolle 1859	Zayü
*Begonianepalensis* (A.DC.) Warb.	Warburg 1894	Cona
*Begoniaovatifolia* A.DC.	de Candolle 1859	Mêdog
*Begoniaoyuniae* M.Taram & N.Krishna	Taram et al. 2020	Mêdog
Begoniaplamatavar.plamata D.Don	Don 1825	Mêdog
Begoniapalmatavar.bowringiana (Champion ex Bentham) Golding & Karegeannes	Golding and Karegeannes 1984	Mêdog
Begoniapalmatavar.khasiana (Irmsch.) Golding & Kareg	Golding and Karegeannes 1984	Mêdog
*Begoniapasighatensis* D.Borah, Taram & Wahlsteen	Borah et al. 2021b	Mêdog
*Begoniapicta* Sm.	Smith 1805	Gyirong, Mêdog, Nyalam
*Begoniapseudoheydei* Y.M.Shui & W.H.Chen	Chen et al. 2019	Mêdog
*Begoniarex* Putz.	Putzey 1857	Mêdog
*Begoniaroxburghii* (Miq.) A.DC.	de Candolle 1864	Mêdog
*Begoniascintillans* Dunn	Dunn 1920	Mêdog
*Begoniashilendrae* Rekha Morris & P.D.McMillan	Morris and McMillan 2012	Cona
Begoniasikkimensisvar.sikkimensisA.DC.	de Candolle 1859	Mêdog
Begoniasikkimensisvar.kamengensis Rekha Morris, P.D.McMillan & Golding ex Golding	Golding 2009	Cona
*Begoniasilletensis* Clarke	Clarke 1879	Mêdog
*Begoniatessaricarpa* C.B.Clarke	Clarke 1879	Mêdog
*Begoniathomsonii* A.DC.	de Candolle 1859	Mêdog
*Begoniaxanthina* Hook.f.	Hooker 1852	Mêdog
*Begoniazhongyangiana* W.G.Wang et S.Z.Zhang	Wang et al. 2019	Mêdog

## Material and methods

### Morphological analysis

The field surveys were conducted on habitat, distribution, population size, morphology and specimen collection of the new species. Diagnosis of the morphological difference between the new species and its similar species was based on literature review, examination of herbarium specimens, and observation of both wild and cultivated plants.

### Phylogenetic analysis

The treatment on sections of *Begonia* follows Shui et al. (2019). To ascertain the relationship of the new species within sect. Platycentrum (Klotzsch) A.DC. (de Candolle 1859), two female and three male individuals were sampled, and three individuals of *B.longifolia*, two individuals of *B.acetosella*, and three individuals of B.acetosellavar.hirtifolia Irmsch. (Irmscher 1939) were sampled and sequenced. 13 taxa within sect. Platycentrum were selected based on Moonlight et al. (2018) to ascertain the phylogenetic relationship of the new species. *Begoniacavaleriei* H.Lév. (Léveille 1909) from sect. Coelocentrum Irmsch. (Irmscher 1939) was used as outgroup. All the voucher specimens were deposited in the herbarium of Xishuangbanna Tropical Botanical Garden, Chinese Academy of Sciences (HITBC). For DNA sequencing, the total genomic DNA was extracted from silica-dried leaves by a modified CTAB protocol (Doyle and Doyle 1987). The chloroplast DNA *rpL16* intron, *ndhA* intron and the nuclear ribosomal DNA internal transcribed spacer (nrITS) region were used to infer the phylogenetic relationship of the new species. The *rpL16* intron were amplified by the primer rpL16-F and rpL16-R and sequenced by the primer rpL16-R and Beg-rpL16 (Chung et al. 2014). For the amplification of the *ndhA* intron the primer *ndhAX*1 and *ndhAX*2 (Thomas et al. 2011) were used. The nrITS region was amplified and sequenced by the primer 51NT and 26S1Rev (Clement et al. 2004). The sampled sequences were downloaded from NCBI and accession numbers were listed in Table 2.

**Table 2.** Sampled taxa and GenBank accession numbers of *Begoniagiganticaulis* and the related taxa used for phylogenetic analysis.

Taxa	Collector, Voucher (Herbarium)	Origin	ITS	*rpL16*	*ndhA*	References
*Begoniaacetosella* Craib	Wang, W.G., WWG004 (HITBC)	Mengla, Yunnan, China	MW690105	MW658199	MW658212	In this study
*B.acetosella* Craib	Wang, W.G., WWG005 (HITBC)	Mengla, Yunnan, China	MW690106	MW658200	MW658213	In this study
B.acetosellavar.hirtifolia Irmsch.	Wang, W.G., WWG0261 (HITBC)	Ruili, Yunnan, China	MW690107	MW658201	—	In this study
B.acetosellavar.hirtifolia Irmsch.	Wang, W.G., WWG0262 (HITBC)	Ruili, Yunnan, China	MW690108	MW658202	MW658214	In this study
B.acetosellavar.hirtifolia Irmsch.	Wang, W.G., WWG0300 (HITBC)	Ruili, Yunnan, China	—	MW658203	MW658215	In this study
*B.aptera* Blume	—	—	AJ491196	—	JF756369	Chiang (unpublised); Thomas et al. (2011)
*B.balansana* Gagnep.	—	—	AF485091	KF707939	MH207051	Forrest and Hollingsworth (2003); Chung et al. (2014); Moonlight et al. (2018)
*B.cathayana* Hemsl.	—	—	AF280106	KF707948	KT599095	Yang et al. (unpublished); Chung et al. (2014); Zhao (unpublished)
*B.cavaleriei* H.Lév.	—	—	KF636430	KF707949	MK548079	Chung et al. (2014); Tong et al. (2019)
*B.decora* Stapf	—	—	KF636435	KF707956	JF756355	Chung et al. (2014); Thomas et al. (2011)
*B.giganticaulis* D.K.Tian & W.G.Wang	Wang, W.G., Li, Y.Y., Ma, X.D. & Shen, J.Y., WWG2014–1 (HITBC)	Mêdog, Tibet, China	MW690097	MW658191	MW658204	In this study
*B.giganticaulis* D.K.Tian & W.G.Wang	Wang, W.G., Li, Y.Y., Ma, X.D. & Shen, J.Y., WWG2015–1 (HITBC)	Mêdog, Tibet, China	MW690098	MW658192	MW658205	In this study
*B.giganticaulis* D.K.Tian & W.G.Wang	Wang, W.G., Li, Y.Y., Ma, X.D. & Shen, J.Y., WWG2015–2 (HITBC)	Mêdog, Tibet, China	MW690099	MW658193	MW658206	In this study
*B.giganticaulis* D.K.Tian & W.G.Wang	Wang, W.G., Li, Y.Y., Ma, X.D. & Shen, J.Y., WWG2014–3 (HITBC)	Mêdog, Tibet, China	MW690100	MW658194	MW658207	In this study
*B.giganticaulis* D.K.Tian & W.G.Wang	Wang, W.G., Li, Y.Y., Ma, X.D. & Shen, J.Y., WWG2014–2 (HITBC)	Mêdog, Tibet, China	MW690101	MW658195	MW658208	In this study
*B.handelii* Irmsch.	—	—	AF485093	KF707969	MH207176	Forrest and Hollingsworth (unpublished); Chung et al. (2014); Moonlight et al. (2018)
*B.hatacoa* Buch.-Ham. ex D.Don	—	—	AF485111	KF707970	JF756354	Forrest and Hollingsworth (unpublished); Chung et al. (2014); Thomas et al. (2011)
*B.longifolia* Blume	Wang, W.G., WWG001 (HITBC)	Mengla, Yunnan, China	MW690102	MW658196	MW658209	In this study
*B.longifolia* Blume	Wang, W.G., WWG002 (HITBC)	Mengla, Yunnan, China	MW690103	MW658197	MW658210	In this study
*B.longifolia* Blume	Wang, W.G., WWG003 (HITBC)	Mengla, Yunnan, China	MW690104	MW658198	MW658211	In this study
*B.nepalensis* (A.DC.) Warb.	—	—	AY753726	—	MH207257	Tebbitt et al. (2006); Moonlight et al. (2018)
*B.obovoidea* Craib	—	—	—	—	JF756386	Thomas et al. (2011)
*B.pavonina* Ridl.	—	—	KF636472	KF708002	JF756356	Chung et al. (2014); Thomas et al. (2011)
*B.pedatifida* H.Lév.	—	—	MK541092	MK548068	MK548115	Tong et al. (2019)
*B.roxburghii* A.DC.	—	—	AF485092	—	JF756371	Forrest and Hollingsworth (2003); Thomas et al. (2011)
*B.versicolor* Irmsch.	—	—	AF485090	KF708023	JF756358	Forrest and Hollingsworth (unpublished); Chung et al. (2014); Thomas et al. (2011)

Sequences of each DNA region were aligned by MUSCLE online (https://www.ebi.ac.uk/Tools/msa/muscle/, Madeira et al. 2019) and adjusted manually when necessary. Indels were treated as gap. For testing the congruence within *rpL16* intron, *ndhA* intron and nrITS, the analysis of the incongruence length difference (ILD) was performed with 100 replicates under default heuristic search using PAUP v.4.0a (Swofford 2002) and the phylogenetic trees were constructed based on each dataset. The p value was 0.40 and no conflict among each phylogenetic trees, indicating the congruence among these datasets (Farris et al. 1994).

The parsimony analysis was conducted using PAUP v.4.0 b10 (Swofford 2002). The Maximum Parsimony (MP) analysis was run using a heuristic search with 1, 000 replicates and tree-bisection-reconnection (TBR) with no reconnection limit. Bootstrap was used to assess the node support by 1000 replicates using TBR branch swapping. The Bayesian analysis was conducted using MrBayes v.3.1.2 (Ronquist and Huelsenbeck 2003) with 1, 000, 000 generations under the Markov chain Monte Carlo (MCMC) chains. The average standard deviation of split frequencies was 0.004210 after 1, 000, 000 generations. The consensus tree was constructed after burn-in 25% of the trees. The Posterior Probability (PP) was used to assess the branch supports.

## Results

### Taxonomic treatment

#### Begonia giganticaulis

Taxon classificationPlantaeCucurbitalesBegoniaceae

D.K.Tian & W.G.Wang sp. nov.

2D720B89-EF53-5D54-88FA-0D47E8F10C53

urn:lsid:ipni.org:names:77234844-1

**Figure 1.**
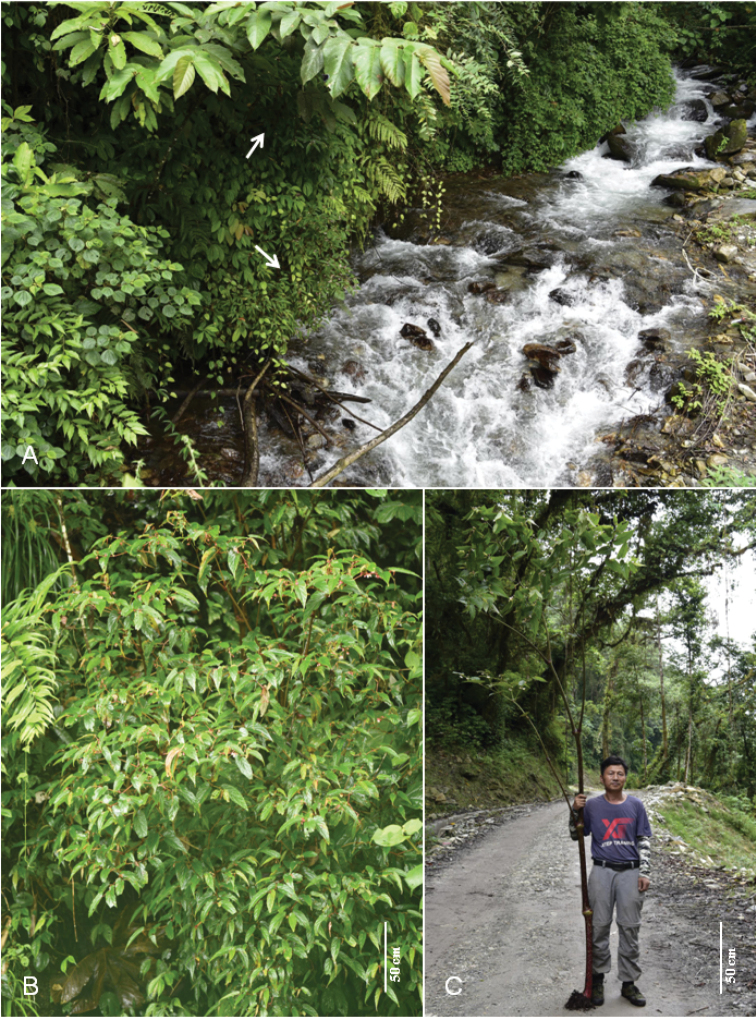
Habitat and large-sized plant of *Begoniagiganticaulis* D.K.Tian & W.G.Wang, sp. nov. **A** habitat showing plants (arrows indicate) growing along stream bank **B** flowering plant growing along slope of valley **C** one of the tallest individuals with Dr. Dai-Ke Tian. (Photos **A** by Dai-Ke Tian **B** by Shi-Wei Guo **C** by Qing-Gong Mao).

**Figure 2.**
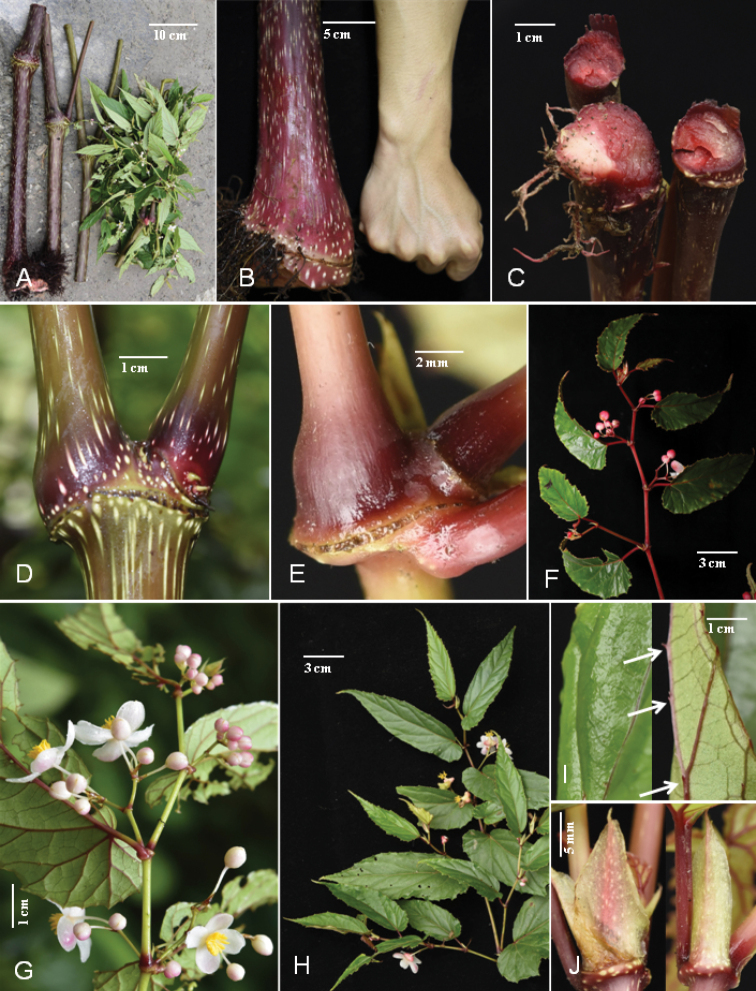
Morphology of *Begoniagiganticaulis* D.K.Tian & W.G.Wang, sp. nov. **A** one of the single tallest plants cut into four sections **B** main stem base **C** stems showing colour of nodal cross-sections **D** main stem with much expanded node and whitish-green lines or spots **E** expanded node on terminal branch **F, G** male plant branches showing inflorescences and different colours **H** female branches **I** adaxially (left) nearly glabrous and abaxially sparse hairs on veins (right, arrows indicate) on blade surfaces **J** stipules showing shape and colour. (Photo **F** by Wen-Guang Wang; others by Dai-Ke Tian).

**Figure 3.**
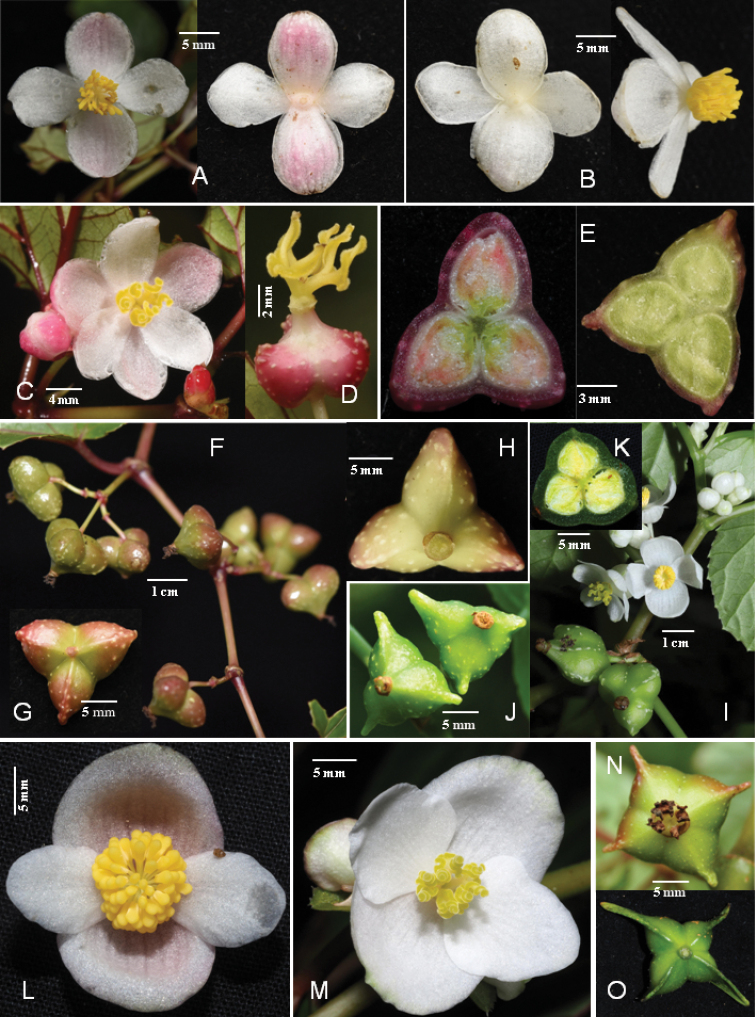
Flower and fruit morphology of *B.giganticaulis* compared with its close species *B.longifolia* and *B.acetosella***A–H***Begoniagiganticaulis***A** staminate flowers with pinkish outer tepals **B** staminate flowers with white tepals **C, D** pistillate flower **E** ovary sections showing different colour **F** fruits on branch **G, H** dorsal and front views of fruits **I–K***B.longifolia***I** flowering and fruiting branch **J** fruits showing short horns **K** ovary dissection **L–O***B.acetosella***L** staminate flower **M** Pistillate flower **N, O** fruits with short horns or wings. (Photos **C** by Shi-Wei Guo **E** (left) **L, M** & **O** by Wen-Guang Wang; others by Dai-Ke Tian).

**Figure 4.**
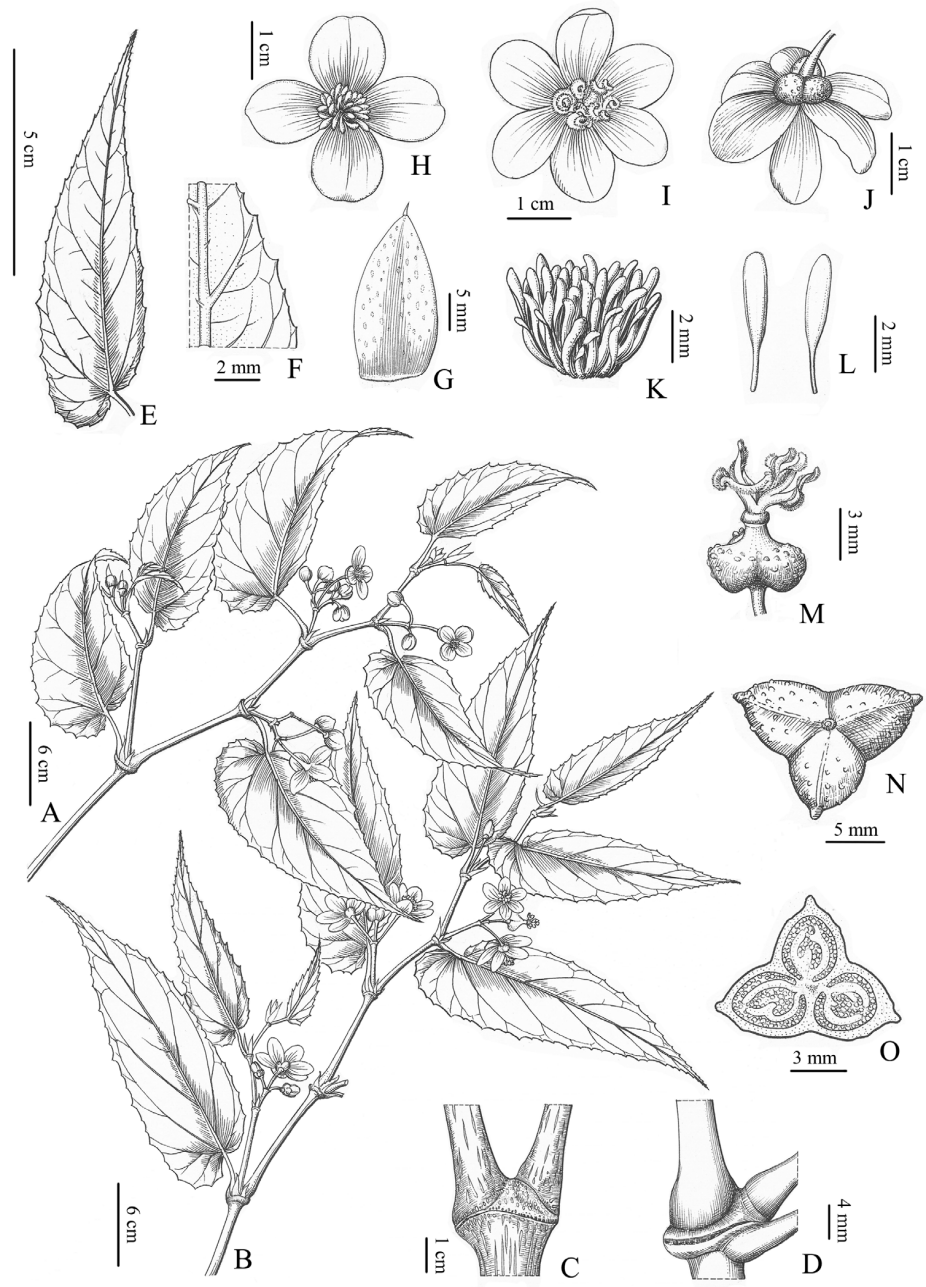
Illustration of *Begoniagiganticaulis* D.K.Tian & W.G.Wang, sp. nov. (Drawn by Mr. Zhi-Min Li) **A** male flowering branches **B** female flowering branches **C** main stem line spots, much expanded node and internode base **D** expanded node and internode base on small upper branches **E** leaf blade **F** leaf (abaxial), showing sparse hairs on veins **G** stipule **H** staminate flowers **I, J** pistillate flower **K** side view of androecium **L** stamens **M** ovary and stigmas **N** fruit **O** dissection of ovary showing placentae.

Figs 1 , 2 , 3 , 4 巨型秋海棠

##### Type.

**China**. Xizang (Tibet) Autonomous Region: Mêdog county (墨脱县), Beibeng town (背崩乡), Baimu Xiri river (白母西日河), forest slope of river valley or water’s edge along stream, 29°21'9"N, 95°11'21"E, elev. 1320 m, 10 September 2020, *Dai-Ke Tian, Fang Wen, Qing-Gong Mao, & Zhu Lu, TDK4773-A* (holotype CSH! Barcode number: 0180561, ♀)

##### Diagnosis.

The new species is mostly similar to *B.longifolia* and *B.acetosella*, but clearly differs from the former mainly by its dioecious (vs. monoecious), taller (to 4 m vs. less than 2 m) plants, longer (vs. shorter) inflorescence, and unique shape of fruits, and differs from the latter mainly by its taller (to 4 m vs. less than 2 m) plants, late and longer (Jun. to Oct. vs. Mar. to Apr.) flowering time, longer (6–20 mm vs. 5–12 mm) pedicel, 6 (vs. 4) tepals of pistillate flower and 3 (vs. 4)-loculed ovary (Table 3, Fig. 3).

**Table 3.** Morphological comparison of *Begoniagiganticaulis*, *B.longifolia* and *B.acetosella*.

Character	*B.giganticaulis*	*B.longifolia*	*B.acetosella*
**Plant**			
sexuality	dioecious	monoecious	dioecious
height (m)	up to 4	less than 2	less than 2
**Petiole** length (cm)	0.7–7	1–12	1–10
**Leaf blade surface**	muriculate	glabrous to less muriculate	muriculate to hirsutulous
**Inflorescence**			
peduncle length (mm)	7–15	4–10	2–10
flower number	1–11	1–11(15)	1–3(5)
**Tepal number of pistillate flower**	6	6	4
**Tepal colour**	pinkish to white	white	pinkish to white
**Ovary**	3-loculed	3-loculed	4-loculed
**Pedicel length** (mm)			
male flower	10–20	5–12	8–12
female flower	6–12	5–12	5–10
**Fruit horn or wing**	none to rarely short crest	none to short crest or horns	short to long horns or wings
**Flowering time**	June-October	June-December	March-April

##### Description.

**Herb** perennial, evergreen, to 4 m tall, dioecious. **Rhizome** short, stout, nearly unbranched, reddish brown, to 12 cm thick. **Stem** erect, reddish brown or green, glabrous, internodes to 5 cm thick, with many longitudinally fusiform whitish spots, cross section of stem often reddish brown, nodes notably enlarged, to 7 cm thick, with unequally oval to round whitish spots, many shrubby branches on the upper part of main stem. **Stipule** long-triangular, light green or pinkish green, 9–25 × 2–8 mm, glabrous, margin entire, dorsal ridge pinkish, apex acuminate with arista 4–6 mm long. **Petioles** green, pink to red, glabrous, 7–22 cm long, 1–3 mm thick. **Leaf blade** ovate-lanceolate to lanceolate, 4–19 × 0.8–8 cm, adaxial green, muriculate to nearly glabrous, adaxial veins slightly concave; abaxial greyish green, veins usually red, convex, main veins sparsely and obliquely strigose; base obliquely cordate, margin shallowly and remotely denticulate, apex long caudate; **Inflorescence** dichasial cyme, axillary, short, 3–5 cm long, unbranched to branched once, rachis glabrous, green, pinkish green to red, base usually red-brown, 7–15 mm long, 1–1.5 mm thick, 3–11 male flowers or 1–5 female per inflorescence. **Bract** often caducous, pinkish green, long triangular, glabrous, ca. 6 × 3 mm, apex acuminate; bracteoles smaller. **Staminate flower**: pedicel glabrous, white, whitish or pinkish green, 10–14 mm long, ca.1 mm thick; corolla 18–24 mm in diameter; tepals 4, subequal, glabrous, outer 2, obovate, 9–14 × 6–9 mm, apex obtuse, adaxially white and middle-upper part abaxially pink, or pure white for some individuals, longitudinal veins unapparent; inner 2, pure white, obovate to obovate-lanceolate, 8–13 × 5–7 mm, apex obtuse; androecium nearly actinomorphic, ca. 5 mm long, 6–7 mm in diam; stamens 48–60, filaments free, 1–2 mm long; anther yellow, 2–3 mm long, apex obtuse or nearly so. **Pistillate flower**: pedicel white or green-white, 6–12 mm long, 0.8–1 mm thick; corolla 20–25 mm, tepals 6, rarely 4, glabrous, outer 3 (rarely 2), obovate or long obovate, thick and rigid, 12–18 × 7–10 mm, adaxial surface nearly white, distinctly concave, abaxially pink on middle-upper part, inner 3 (rarely 2), obovate-lanceolate to oblanceolate or long elliptical, slightly narrower than outer tepals, 10–19 × 6–8 mm, white, glabrous, apex obtuse; styles + stigmas 5 mm long, 7–8 mm wide; styles 3, free; stigmas yellow, nearly U-shaped, each side spirally twisted 1.5 circles; ovary pink or green, with white convex spots; placentation axile, 3-loculed, each placenta 2-branched. **Peduncle** green to pinkish green, glabrous, 8–12 mm long, ca. 1 mm thick. **Fruit** red, pink or green, glabrous, triangular-gyroscopic, 8–11 × 1–12 mm wide, concave between two placentas, wingless to occasionally short ridged, apex with beak 3–4 mm long. Flowering Jun.–Oct., fruiting Jul.–Dec.

##### Additional specimen examined.

**China. Xizang**: Mêdog County (墨脱县), Beibeng Town (背崩乡), Baimu Xiri River (白母西日河), forest slope of river valley or water’s edge along stream, 29°21'9"N, 95°11'21"E, elev. 1320 m, 10 September 2020, *Dai-Ke Tian, Fang Wen, Qing-Gong Mao, & Zhu Lu TDK4773-B* (paratype CSH!, ♂); 29°20'0"N, 95°10'49"E, elev. 1110 m, 10 September 2020, *Dai-Ke Tian, Fang Wen, Qing-Gong Mao, & Zhu Lu TDK4765-A, TDK4765-B*, (paratype CSH!); 29°18'32"N, 95°10'38"E, elev. 980 m, 10 September 2020, *Dai-Ke Tian, Fang Wen, Qing-Gong Mao, & Zhu Lu TDK4777* (paratype CSH!); near Ani Bridge (阿尼桥), 29°17'8.41"N, 95°10'3.23"E, elev. 810 m, 3 July 2020, *Wen-Guang Wang, You-Yun Li, Xing-Da Ma, & Jian-Yong Shen, WWG 2014* (paratype, HITBC!), *WWG 2015* (paratype HITBC!); elev. 1100 m, 16 September 1974, *anonymous 2608* (paratype PE!); elev. 800–1400 m, 30 June 1980, *Wei-Lie Chen 10809* (PE!); near No. 2 Bridge, 29°16'42"N, 95°10'49"E, elev. 810 m, 1 October 2017, *Dai-Ke Tian, Yan Xiao, Xin Zhong, Li-Zhi Tian & Zhu Lu TDK3429* (paratype CSH!); Beibeng to Hanmi (汗密), elev. 840 m, 7 August 2010, South Tibet Expedition Team (藏南队), *Xiao-Hua Jin, Shu-Dong Zhang, Zhong-Yang Li, Bao-Cheng Wu, Xian-Yun Mu, Jing Li & Wei-Tao Jin, STET2304* (paratype PE!); Hanmi to Maniweng (马尼翁), elev. 800–1000 m, 6 August 1974, *Qingzang Team 74-4114* (paratype PE!); elev. 1200 m, 24 June 1983, *Bo-Sheng Li & Shu-Zhi Chen 05229* (paratype PE!); Maniweng to Ani Bridge, elev. 700–1000 m, 3 August 1972, *Tibet Expedition Team, Institute of Biology 1631* (paratype HNWP!).

##### Distribution and habitat.

**Figure 5.**
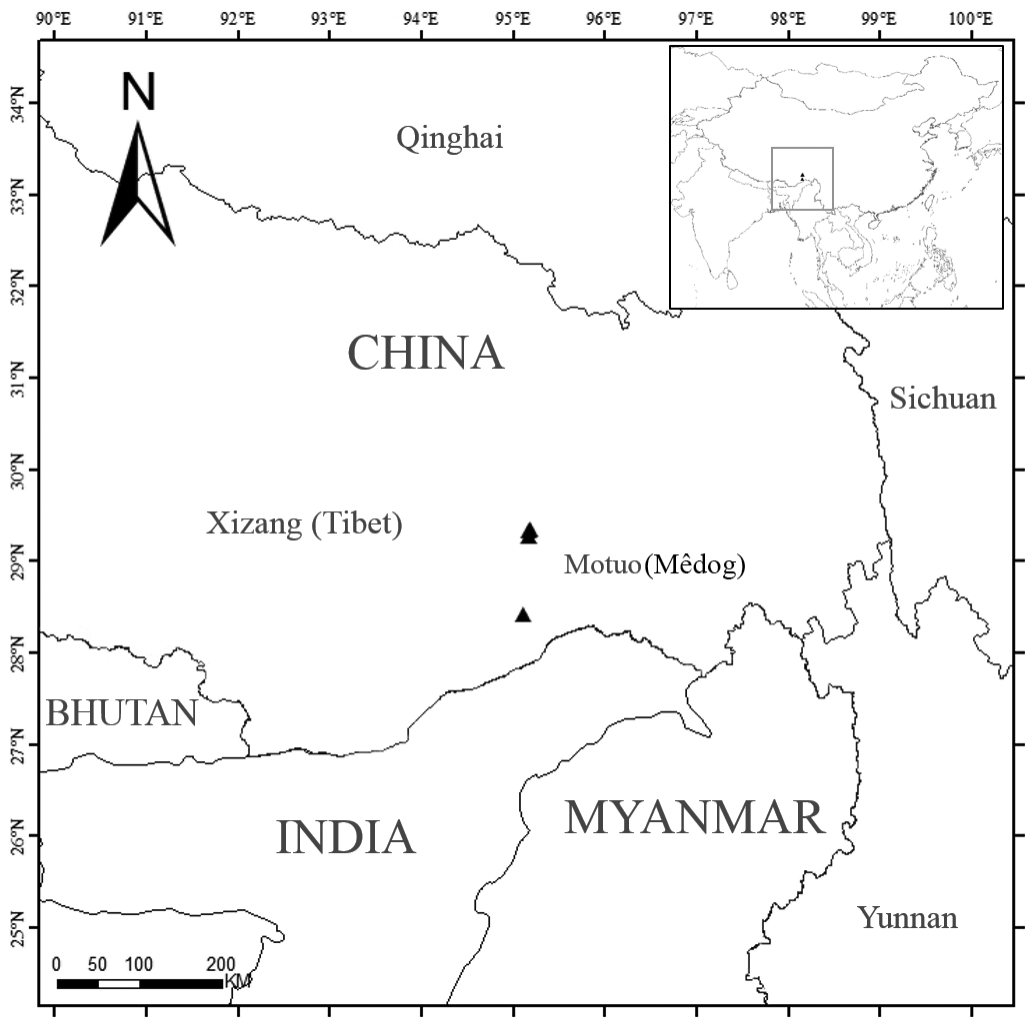
Distribution of *B.giganticaulis* (triangles) from southern Xizang, China.

Currently known from at least two localities in Mêdog, southern Xizang (Tibet), China (Fig. 5). It grows on the slopes under forest along streams, elevation 450–1400 m.

##### Conservation status.

*Begoniagiganticaulis* is currently found in at least two localities in Mêdog of Tibet. Additional populations might be discovered when more surveys are conducted in China-India border region. However, based on current data, it should be categorised as Endangered: B2a (IUCN 2019) due to < 500 km^2^ area of occupancy with severely fragmented habitat consisting of < 5 known populations totally under 1000 individuals by estimation.

##### Etymology.

The specific epithet refers to the huge (very tall and thick stem) plant size of the new species, which is the tallest begonia in Asia.

### Molecular systematic relationship

We obtained 12 nrITS, 13 *rpL16* intron, and 13 *ndhA* intron of the new species and related *Begonia* taxa. In order to reconstruct the phylogenetic relationship of the new species, 13 taxa within sect. Platycentrum were included and *B.cavaleriei* from sect. Coelocentrum was selected as outgroup. In total, the matrix was composed of 26 accessions and contained the 962 bp *rpL16* intron, the 1109 bp *ndhA* intron and the 672 bp nrITS sequence. Of the total 2743 characters, 132 were parsimony informative.

**Figure 6.**
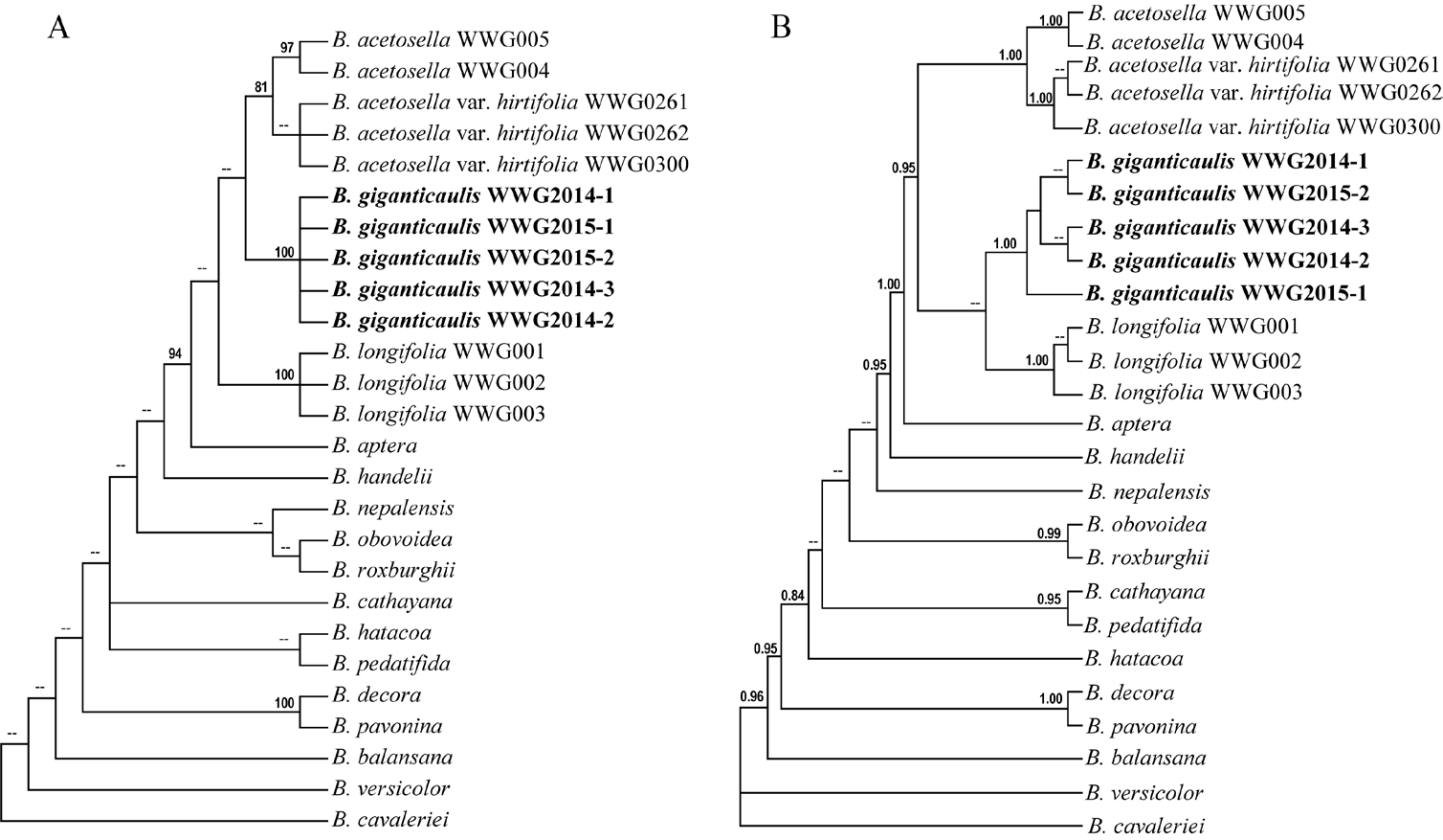
Phylogenetic tree inferred by MP**A** and BI **B** analyses based on the combined matrix of two plastid loci (*rpL16* intron and *ndhA* intron) and nuclear ITS. Maximum parsimony bootstrap **A** and Bayesian inference posterior probability values **B** are labelled on the branches; when the number is below 80 and 0.80 in maximum parsimony bootstrap and Bayesian inference posterior probability, respectively, the branches are labelled—.

**Notes.** – The earliest specimen of *Begoniagiganticaulis* was collected in 1972 between Maliweng and Ani Bridge, Mêdog, Tibet, China. This species is similar to *B.acetosella* in appearance when its flowers are unavailable for observation, therefore, it was misidentified (24 June 1983, *Bo-Sheng Li & Shu-Zhi Chen 05229*, PE! was wrongly identified as *B.acetosella* by C.Z. Gu in March 2004). Also, due to its high similarity to *B.longifolia* particularly in morphology of flowers and fruits, *B.giganticaulis* was wrongly labelled as *B.longifolia* by Morris (2010) who found this species in southern Mêdog county.

Based on MP analysis, the new species was clustered with *B.acetosella* and B.acetosellavar.hirtifolia (Fig. 6A), while it was clustered with *B.longifolia* under BI analysis (Fig. 6B). Both MP and BI analyses showed that all five individuals of the new species were clustered together and separated from other taxa (Fig. 6A, BS: 100%; Fig. 6B, PP:1.00).

## Supplementary Material

XML Treatment for Begonia giganticaulis
